# *N*-(3-Chloro­phen­yl)succinamic acid

**DOI:** 10.1107/S1600536810008949

**Published:** 2010-03-17

**Authors:** B. Thimme Gowda, Sabine Foro, B. S. Saraswathi, Hartmut Fuess

**Affiliations:** a Department of Chemistry, Mangalore University, Mangalagangotri 574 199, Mangalore, India; b Institute of Materials Science, Darmstadt University of Technology, Petersenstrasse 23, D-64287 Darmstadt, Germany

## Abstract

In the title compound, C_10_H_10_ClNO_3_, the N—H and C=O bonds in the amide segment are *trans* to each other. In the crystal structure, the mol­ecules are linked into infinite chains through inter­molecular N—H⋯O and O—H⋯O hydrogen bonds.

## Related literature

For our study of the effect of ring and side-chain substitutions on the structures of anilides and for related structures, see: Gowda *et al.* (2009**a* ▶,b* ▶; 2010 ▶); Jagannathan *et al.* (1994 ▶).
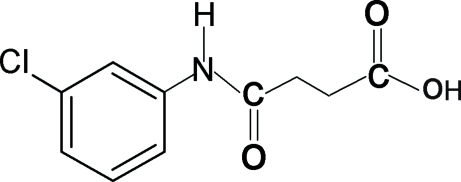


## Experimental

### 

#### Crystal data

C_10_H_10_ClNO_3_
*M* *_r_* = 227.64
Orthorhombic, 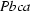

*a* = 10.0308 (8) Å
*b* = 11.1810 (9) Å
*c* = 19.036 (2) Å
*V* = 2135.0 (3) Å^3^
*Z* = 8
Mo *K*α radiation
μ = 0.34 mm^−1^
*T* = 299 K
0.24 × 0.20 × 0.06 mm

#### Data collection

Oxford Diffraction Xcalibur diffractometer with a Sapphire CCD detector
Absorption correction: multi-scan (*CrysAlis RED*; Oxford Diffraction, 2009 ▶) *T*_min_ = 0.922, *T*_max_ = 0.980
8200 measured reflections
2184 independent reflections
1137 reflections with *I* > 2σ(*I*)
*R*_int_ = 0.045

#### Refinement

*R*[*F* ^2^ > 2σ(*F* ^2^)] = 0.058
*wR*(*F* ^2^) = 0.152
*S* = 1.02
2184 reflections
142 parameters
2 restraints
H atoms treated by a mixture of independent and constrained refinement
Δρ_max_ = 0.30 e Å^−3^
Δρ_min_ = −0.39 e Å^−3^

### 

Data collection: *CrysAlis CCD* (Oxford Diffraction, 2009 ▶); cell refinement: *CrysAlis RED* (Oxford Diffraction, 2009 ▶); data reduction: *CrysAlis RED*; program(s) used to solve structure: *SHELXS97* (Sheldrick, 2008 ▶); program(s) used to refine structure: *SHELXL97* (Sheldrick, 2008 ▶); molecular graphics: *PLATON* (Spek, 2009 ▶); software used to prepare material for publication: *SHELXL97*.

## Supplementary Material

Crystal structure: contains datablocks I, global. DOI: 10.1107/S1600536810008949/bt5210sup1.cif

Structure factors: contains datablocks I. DOI: 10.1107/S1600536810008949/bt5210Isup2.hkl

Additional supplementary materials:  crystallographic information; 3D view; checkCIF report

## Figures and Tables

**Table 1:** Hydrogen-bond geometry (Å, °)

*D*—H⋯*A*	*D*—H	H⋯*A*	*D*⋯*A*	*D*—H⋯*A*
O3—H3*O*⋯O1^i^	0.82 (2)	1.92 (2)	2.693 (3)	158 (5)
N1—H1*N*⋯O2^ii^	0.85 (2)	2.02 (2)	2.872 (4)	173 (3)

Symmetry codes: (i) 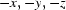; (ii) 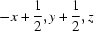.

**Fig. 1.**
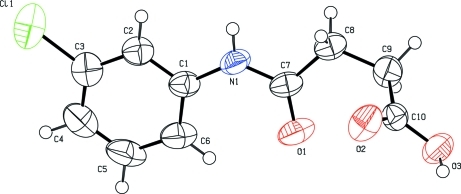
Molecular structure of the title compound, showing the atom labelling scheme. The displacement ellipsoids are drawn at the 50% probability level. The H atoms are represented as small spheres of arbitrary radii.

**Fig. 2.**
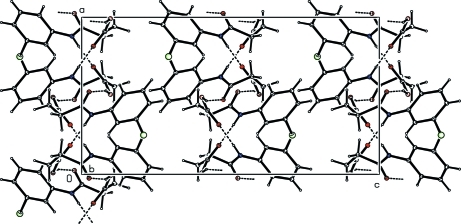
Molecular packing of the title compound with hydrogen bonding shown as dashed lines.

## supplementary crystallographic information

### Comment

As a part of studying the effect of ring and side chain substitutions on the structures of anilides (Gowda *et al.*, 2009*a,b*; 2010), the crystal structure of *N*-(3-chlorophenyl)succinamic acid (I) has been determined. The conformations of N—H and C=O bonds in the amide segment are *anti* to each other, similar to those observed in *N*-(2-chlorophenyl)succinamic acid (II)(Gowda *et al.*, 2009*b*) and *N*-(4-chlorophenyl)succinamic acid (III) (Gowda *et al.*, 2009*a*) and *N*-(3-methylphenyl)succinamic acid (IV)(Gowda *et al.*, 2010). But the conformation of the amide oxygen and the carbonyl oxygen of the acid segment are *syn* to each other, similar to that observed in (IV), but contrary contrary to the *anti* conformation observed in (II) and (III). Further, the conformation of both the C=O bonds are *anti* to the H atoms of their adjacent –CH_2_ groups (Fig. 1) and the C=O and O—H bonds of the acid group are in *syn* position to each other, similar to that observed in (II), (III) and (IV).

The conformation of the amide hydrogen is *syn* to the *meta*- Cl group in the benzene ring, similar to that of the *ortho*-Cl in (II), but contrary to the *anti* conformation observed between the amide hydrogen and the *meta*-methyl group in (IV).

The N—H···O and O—H···O intermolecular hydrogen bonds pack the mpolecules into infinite chains in the structure (Table 1, Fig.2).

The packing of molecules involving dimeric hydrogen bonded association of each carboxyl group with a centrosymmetrically related neighbor has also been observed (Jagannathan *et al.*, 1994).

### Experimental

The solution of succinic anhydride (0.01 mole) in toluene (25 ml) was treated dropwise with the solution of *m*-chloroaniline (0.01 mole) also in toluene (20 ml) with constant stirring. The resulting mixture was stirred for about one h and set aside for an additional hour at room temperature for completion of the reaction. The mixture was then treated with dilute hydrochloric acid to remove the unreacted *m*-chloroaniline. The resultant solid *N*-(3-chlorophenyl)succinamic acid was filtered under suction and washed thoroughly with water to remove the unreacted succinic anhydride and succinic acid. It was recrystallized to constant melting point from ethanol.

The purity of the compound was checked by elemental analysis and characterized by its infrared and NMR spectra. The plate like colorless single crystals used in X-ray diffraction studies were grown in ethanolic solution by slow evaporation at room temperature.

### Refinement

The H atoms of the OH and NH group were located in a difference map and refined with a distance restraint of O—H = 0.82 (2) %A and N—H = 0.86 (2) %A. The other H atoms were positioned with idealized geometry using a riding model with C—H = 0.93–0.97 Å. All H atoms were refined with isotropic displacement parameters  set to 1.2 times of the *U*_eq_ of the parent atom.

### Crystal data

**Table:**

C_10_H_10_ClNO_3_	*F*(000) = 944
*M**_r_* = 227.64	*D*_x_ = 1.416 Mg m^−^^3^
Orthorhombic, *P**b**c**a*	Mo *K*α radiation, λ = 0.71073 Å
Hall symbol: -P 2ac 2ab	Cell parameters from 2016 reflections
*a* = 10.0308 (8) Å	θ = 2.7–27.7°
*b* = 11.1810 (9) Å	µ = 0.34 mm^−^^1^
*c* = 19.036 (2) Å	*T* = 299 K
*V* = 2135.0 (3) Å^3^	Plate, colourless
*Z* = 8	0.24 × 0.20 × 0.06 mm

### Data collection

**Table:**

Oxford Diffraction Xcalibur diffractometer with a Sapphire CCD detector	2184 independent reflections
Radiation source: fine-focus sealed tube	1137 reflections with *I* > 2σ(*I*)
graphite	*R*_int_ = 0.045
Rotation method data acquisition using ω and φ scans.	θ_max_ = 26.4°, θ_min_ = 2.9°
Absorption correction: multi-scan (*CrysAlis RED*; Oxford Diffraction, 2009)	*h* = −9→12
*T*_min_ = 0.922, *T*_max_ = 0.980	*k* = −12→13
8200 measured reflections	*l* = −22→23

### Refinement

**Table:**

Refinement on *F*^2^	Primary atom site location: structure-invariant direct methods
Least-squares matrix: full	Secondary atom site location: difference Fourier map
*R*[*F*^2^ > 2σ(*F*^2^)] = 0.058	Hydrogen site location: inferred from neighbouring sites
*wR*(*F*^2^) = 0.152	H atoms treated by a mixture of independent and constrained refinement
*S* = 1.02	*w* = 1/[σ^2^(*F*_o_^2^) + (0.0603*P*)^2^ + 1.1737*P*] where *P* = (*F*_o_^2^ + 2*F*_c_^2^)/3
2184 reflections	(Δ/σ)_max_ = 0.012
142 parameters	Δρ_max_ = 0.30 e Å^−^^3^
2 restraints	Δρ_min_ = −0.39 e Å^−^^3^

### Special details

**Table:**

Experimental. CrysAlis RED (Oxford Diffraction, 2009) Empirical absorption correction using spherical harmonics, implemented in SCALE3 ABSPACK scaling algorithm.
Geometry. All esds (except the esd in the dihedral angle between two l.s. planes) are estimated using the full covariance matrix. The cell esds are taken into account individually in the estimation of esds in distances, angles and torsion angles; correlations between esds in cell parameters are only used when they are defined by crystal symmetry. An approximate (isotropic) treatment of cell esds is used for estimating esds involving l.s. planes.
Refinement. Refinement of *F*^2^ against ALL reflections. The weighted *R*-factor wR and goodness of fit *S* are based on *F*^2^, conventional *R*-factors *R* are based on *F*, with *F* set to zero for negative *F*^2^. The threshold expression of *F*^2^ > σ(*F*^2^) is used only for calculating *R*-factors(gt) etc. and is not relevant to the choice of reflections for refinement. *R*-factors based on *F*^2^ are statistically about twice as large as those based on *F*, and *R*- factors based on ALL data will be even larger.

### Fractional atomic coordinates and isotropic or equivalent isotropic displacement parameters (Å^2^)

**Table:**

	*x*	*y*	*z*	*U*_iso_*/*U*_eq_	
Cl1	0.25030 (11)	0.71276 (10)	0.20800 (6)	0.0858 (4)	
O1	−0.0249 (2)	0.23883 (19)	0.02471 (13)	0.0655 (7)	
O2	0.1845 (3)	0.0367 (2)	−0.03891 (14)	0.0673 (7)	
O3	0.0091 (3)	−0.0318 (2)	−0.09646 (13)	0.0651 (7)	
H3O	0.033 (5)	−0.097 (2)	−0.081 (2)	0.098*	
N1	0.1200 (3)	0.3941 (2)	0.03276 (15)	0.0539 (8)	
H1N	0.181 (3)	0.431 (3)	0.0101 (16)	0.065*	
C1	0.0900 (3)	0.4445 (3)	0.09884 (18)	0.0484 (8)	
C2	0.1706 (4)	0.5390 (3)	0.12010 (18)	0.0523 (9)	
H2	0.2395	0.5654	0.0913	0.063*	
C3	0.1482 (4)	0.5930 (3)	0.1835 (2)	0.0574 (10)	
C4	0.0471 (5)	0.5568 (4)	0.2270 (2)	0.0714 (12)	
H4	0.0329	0.5943	0.2700	0.086*	
C5	−0.0325 (5)	0.4644 (4)	0.2058 (2)	0.0741 (12)	
H5	−0.1015	0.4393	0.2349	0.089*	
C6	−0.0129 (4)	0.4072 (3)	0.1420 (2)	0.0627 (10)	
H6	−0.0682	0.3446	0.1284	0.075*	
C7	0.0649 (3)	0.2997 (3)	−0.00050 (19)	0.0480 (8)	
C8	0.1239 (3)	0.2756 (3)	−0.07174 (17)	0.0519 (9)	
H8A	0.2183	0.2590	−0.0664	0.062*	
H8B	0.1151	0.3468	−0.1004	0.062*	
C9	0.0587 (4)	0.1716 (3)	−0.10939 (18)	0.0566 (9)	
H9A	−0.0373	0.1824	−0.1086	0.068*	
H9B	0.0870	0.1716	−0.1581	0.068*	
C10	0.0920 (4)	0.0530 (3)	−0.07727 (17)	0.0444 (8)	

### Atomic displacement parameters (Å^2^)

**Table:**

	*U*^11^	*U*^22^	*U*^33^	*U*^12^	*U*^13^	*U*^23^
Cl1	0.0739 (7)	0.0821 (8)	0.1014 (9)	0.0073 (6)	−0.0183 (7)	−0.0295 (6)
O1	0.0610 (15)	0.0445 (13)	0.091 (2)	−0.0112 (12)	0.0190 (14)	0.0046 (12)
O2	0.0650 (17)	0.0477 (14)	0.0894 (19)	0.0055 (13)	−0.0291 (16)	0.0059 (13)
O3	0.0739 (18)	0.0504 (14)	0.0712 (17)	−0.0174 (15)	−0.0159 (14)	0.0058 (13)
N1	0.0531 (18)	0.0452 (16)	0.063 (2)	−0.0146 (14)	0.0162 (15)	−0.0029 (14)
C1	0.049 (2)	0.0408 (18)	0.055 (2)	0.0040 (16)	0.0087 (18)	0.0059 (16)
C2	0.045 (2)	0.054 (2)	0.058 (2)	0.0041 (18)	0.0071 (17)	0.0025 (17)
C3	0.053 (2)	0.057 (2)	0.062 (2)	0.0130 (18)	−0.009 (2)	0.0002 (19)
C4	0.088 (3)	0.075 (3)	0.052 (3)	0.019 (3)	0.008 (2)	0.004 (2)
C5	0.083 (3)	0.074 (3)	0.066 (3)	0.007 (3)	0.032 (2)	0.018 (2)
C6	0.060 (2)	0.054 (2)	0.074 (3)	−0.0032 (19)	0.018 (2)	0.0106 (19)
C7	0.0447 (18)	0.0346 (16)	0.065 (2)	0.0046 (15)	0.0048 (19)	0.0118 (16)
C8	0.056 (2)	0.0356 (17)	0.064 (2)	0.0035 (16)	0.0013 (19)	0.0076 (16)
C9	0.065 (2)	0.0490 (19)	0.056 (2)	0.0029 (18)	−0.0134 (19)	0.0037 (17)
C10	0.047 (2)	0.0430 (19)	0.0430 (19)	0.0012 (16)	0.0024 (17)	−0.0039 (15)

### Geometric parameters (Å, °)

**Table:**

Cl1—C3	1.749 (4)	C4—C5	1.366 (6)
O1—C7	1.226 (4)	C4—H4	0.9300
O2—C10	1.195 (4)	C5—C6	1.388 (5)
O3—C10	1.313 (4)	C5—H5	0.9300
O3—H3O	0.820 (19)	C6—H6	0.9300
N1—C7	1.349 (4)	C7—C8	1.504 (5)
N1—C1	1.411 (4)	C8—C9	1.515 (4)
N1—H1N	0.853 (18)	C8—H8A	0.9700
C1—C6	1.384 (5)	C8—H8B	0.9700
C1—C2	1.391 (5)	C9—C10	1.498 (4)
C2—C3	1.369 (5)	C9—H9A	0.9700
C2—H2	0.9300	C9—H9B	0.9700
C3—C4	1.371 (5)		
			
C10—O3—H3O	111 (3)	C1—C6—H6	120.4
C7—N1—C1	130.1 (3)	C5—C6—H6	120.4
C7—N1—H1N	116 (2)	O1—C7—N1	123.5 (3)
C1—N1—H1N	114 (2)	O1—C7—C8	122.8 (3)
C6—C1—C2	119.4 (3)	N1—C7—C8	113.7 (3)
C6—C1—N1	124.6 (3)	C7—C8—C9	113.2 (3)
C2—C1—N1	116.0 (3)	C7—C8—H8A	108.9
C3—C2—C1	119.8 (3)	C9—C8—H8A	108.9
C3—C2—H2	120.1	C7—C8—H8B	108.9
C1—C2—H2	120.1	C9—C8—H8B	108.9
C2—C3—C4	121.6 (4)	H8A—C8—H8B	107.7
C2—C3—Cl1	118.5 (3)	C10—C9—C8	112.9 (3)
C4—C3—Cl1	119.9 (3)	C10—C9—H9A	109.0
C5—C4—C3	118.5 (4)	C8—C9—H9A	109.0
C5—C4—H4	120.7	C10—C9—H9B	109.0
C3—C4—H4	120.7	C8—C9—H9B	109.0
C4—C5—C6	121.7 (4)	H9A—C9—H9B	107.8
C4—C5—H5	119.2	O2—C10—O3	123.5 (3)
C6—C5—H5	119.2	O2—C10—C9	123.9 (3)
C1—C6—C5	119.1 (4)	O3—C10—C9	112.6 (3)
			
C7—N1—C1—C6	−4.0 (6)	N1—C1—C6—C5	−179.8 (3)
C7—N1—C1—C2	176.7 (3)	C4—C5—C6—C1	0.2 (6)
C6—C1—C2—C3	0.6 (5)	C1—N1—C7—O1	−1.2 (5)
N1—C1—C2—C3	179.9 (3)	C1—N1—C7—C8	178.9 (3)
C1—C2—C3—C4	−0.4 (5)	O1—C7—C8—C9	1.7 (4)
C1—C2—C3—Cl1	−179.2 (3)	N1—C7—C8—C9	−178.4 (3)
C2—C3—C4—C5	0.0 (6)	C7—C8—C9—C10	−71.0 (4)
Cl1—C3—C4—C5	178.8 (3)	C8—C9—C10—O2	−18.4 (5)
C3—C4—C5—C6	0.1 (6)	C8—C9—C10—O3	162.3 (3)
C2—C1—C6—C5	−0.5 (5)		

### Hydrogen-bond geometry (Å, °)

**Table:**

*D*—H···*A*	*D*—H	H···*A*	*D*···*A*	*D*—H···*A*
O3—H3O···O1^i^	0.82 (2)	1.92 (2)	2.693 (3)	158 (5)
N1—H1N···O2^ii^	0.85 (2)	2.02 (2)	2.872 (4)	173 (3)

Symmetry codes: (i) −*x*, −*y*, −*z*; (ii) −*x*+1/2, *y*+1/2, *z*.
